# Sedation assessment in a mobile intensive care unit: a prospective pilot-study on the relation of clinical sedation scales and the bispectral index

**DOI:** 10.1186/s13054-014-0615-9

**Published:** 2014-11-24

**Authors:** Johannes Prottengeier, Andreas Moritz, Sebastian Heinrich, Christine Gall, Joachim Schmidt

**Affiliations:** Department of Anaesthesiology, Erlangen University Hospital, Krankenhausstrasse 12, 91054 Erlangen, Germany; Department of Medical Informatics, Biometry and Epidemiology, Waldstrasse 6, 91054 Erlangen, Germany

## Abstract

**Introduction:**

The critically-ill undergoing inter-hospital transfers commonly receive sedatives in continuation of their therapeutic regime or to facilitate a safe transfer shielded from external stressors. While sedation assessment is well established in critical care in general, there is only little data available relating to the special conditions during patient transport and their effect on patient sedation levels. The aim of this prospective study was to investigate the feasibility and relationship of clinical sedation assessment (Richmond Agitation-Sedation Scale (RASS)) and objective physiological monitoring (bispectral index (BIS)) during patient transfers in our Mobile-ICU.

**Methods:**

The levels of sedation of 30 pharmacologically sedated patients were evaluated at 12 to 17 distinct measurement points spread strategically over the course of a transfer by use of the RASS and BIS. To investigate the relation between the RASS and the BIS, Spearman’s squared rank correlation coefficient (ρ^2^) and the Kendall’s rank correlation coefficient (τ) were calculated. The diagnostic value of the BIS with respect to the RASS was investigated by its sensitivity and positive predictive value for possible patient awakening. Therefore, measurements were dichotomized considering a clinically sensible threshold of 80 for BIS-values and classifying RASS values being nonnegative.

**Results:**

Spearman’s rank correlation resulted to ρ^2^ = 0.431 (confidence interval (CI) = 0.341 to 0.513). The Kendall’s correlation coefficient was calculated as τ = 0.522 (CI = 0.459 to 0.576). Awakening of patients (RASS ≥0) was detected by a BIS value of 80 and above with a sensitivity of 0.97 (CI = 0.89 to 1.00) and a positive predictive value of 0.59 (CI = 0.45 to 0.71).

**Conclusions:**

Our study demonstrates that the BIS-Monitor can be used for the assessment of sedation levels in the intricate environment of a Mobile-ICU, especially when well-established clinical scores as the RASS are impracticable. The use of BIS is highly sensitive in the detection of unwanted awakening of patients during transfers.

**Electronic supplementary material:**

The online version of this article (doi:10.1186/s13054-014-0615-9) contains supplementary material, which is available to authorized users.

## Introduction

The transfer of critically ill patients in specialized mobile intensive care units (mobile ICU) has become a common procedure in times of czentralization of tertiary care [1]. In Germany a large proportion of such transfers are conducted as ground transport by a dedicated team consisting of an intensive care physician accompanied by two specially trained paramedics utilizing an ambulance truck that provides standard ICU bed-space facilities. Maintaining the high levels of care as exerted on both the referring and the accepting ICU is the major goal of such transfers [2,3]. Many of the patients undergoing such transfers are receiving sedatives either as a part of their ongoing therapeutic regimen or as a supplement to facilitate a safe transfer shielded from external stressors [4].

In the ICU setting there are well-established practice guidelines in place to assure adequate levels of sedation and a valid assessment thereof [5]: it is recommended to titrate sedatives towards a level of light sedation. Besides the obvious ethical obligation to provide adequate analgesia and sedation for painful and stressing procedures, there is evidence of the negative effects of both: too deep and too shallow levels of sedation [6,7]. Clinical sedation scales such as the Richmond agitation-sedation scale (RASS) have proven themselves to be reliable assessment tools in the ICU context [8]. Neurophysiological monitors like the bispectral index (BIS) are suggested as adjunct measures for clinical settings where subjective methods are unobtainable [5]. These monitors have shown good correlation with established clinical scores [9–11]. The aim of this study was to analyse the feasibility of BIS monitoring in a mobile ICU, its relationship to the principal clinical tool of sedation assessment, the RASS, and especially the value of BIS in the detection of patient awakening. In addition we investigated the possible need for such an extended range of patient monitoring as we identified challenges and limitations of mobile ICU transfers in relationship to patient observation.

## Materials and methods

### Patients

This prospective study was undertaken by the mobile ICU service stationed at the Erlangen University Hospital, which is handling approximately 700 ICU transfers per year. The University of Erlangen-Nuremberg ethics committee approved this study and waived the need for formal informed consent from the patients’ legal guardians or relatives due to the urgent nature of the retrieval missions of the critically ill and the purely observational nature of our study (Reg number 328_12 B). All patients with a known loss of hearing, higher cerebral dysfunctions (neurotauma, apoplexia, hypoxic brain damage), those receiving ketamine, and hemodynamically instable patients were excluded in accordance to the study protocol. Abort criteria included all scenarios where patient care was potentially or actually hindered by our investigation (Table 1). A total of 30 pharmacologically sedated adult patients undergoing interhospital transfer were enrolled over a period of 3 months.

**Table 1:** **Exclusion criteria**

**Patient factors**	**Iatrogenic factors**	**General criteria**
Legal minor (age < 18 years)	Ketamine sedation	Critical incidents
Degenerative muscle disorders	Neuromuscular Blockage	Impairment of patient care
Cerebral impairment		
- Hypoxia/Apoplexia		
- Cerebral bleeding		
- Neurotrauma		
- Brain-Tumors		
- Recent CNS surgery		
- Loss or difficulty of hearing		
- Tremor		
Hemodynamic instability		

### Materials

The Erlangen mobile ICU is a medium-sized Mercedes Atego® truck with a custom-built cargo box, containing equipment equivalent to a standard ICU bed space. In addition to the regular configuration a two-lead BIS-Vista® Monitor (Covidien, Mansfield, MA, USA) was installed on board (the arrangement can be seen in Additional file 1).

To determine possible sources of irritation for patient monitoring we conducted noise and vibration measurements during exemplary drives in our mobile ICU. Noise levels were recorded with an Integrating Sound Level Meter - Type 2239A (Brüel & Kjær, Nærum, Denmark) placed in the centre of the patient transport compartment. Measurements of noise exposure were taken on a single day with exemplary transfer routes. The exposure to vibrations was measured with a Triaxial Human Vibration Meter VM30-H (Metra Mess- und Frequenztechnik, Radebeul, Germany) placed underneath the patient’s central thorax upon the mattress of the transport stretcher.

### Methods

We investigated the subjective perception of challenges and limitations as they were experienced by the escorting physician through means of a nine-item questionnaire for each transfer. During the patient-centred part of the study, the accompanying intensivists did not partake in the assessment of sedation by RASS or BIS and were blinded to these findings, allowing for the strictly observational character of our study. It was the physicians’ assignment to subjectively comment on general aspects of the particular transfer by means of answering a questionnaire addressing items in relation to the job at hand. For sedation assessment, all paramedics were trained in obtaining the RASS and BIS measures. BIS data were excluded from sedation assessment if the BIS monitor electromyography index was >50% and/or if the signal quality index fell short of 75%. Those poor-quality data points were registered as artefacts in our study protocol.

The levels of sedation of each patient were evaluated at 12 to 17 distinct measurement points (Table 2) spread strategically over the course of a transfer by use of the RASS and the BIS. RASS is a 10-level single-item numerical scale deduced from response to auditory and physical stimulation and patient observation (+4 combative to -5 unarousable). BIS is a dimensionless numerical parameter that is derived from algorithmic analysis of a patient’s electroencephalogram and is widely used to determine levels of anaesthesia during surgery. A range of 40 to 60 is commonly considered to reflect an appropriate level of anaesthesia, while values above 80 may indicate patient awakening.

**Table 2:** **Distribution of measurement points**

**MeasurementNo.**	**Phase of transfer**
1	First contact with patient
2	Disconnection from stationary ICU-supply
3	Bed-to-stretcher transfer
4	Transport inside referring hospital
5	Loading
6	Start mobile ICU
7	Intra-urban 1
8	Inter-urban transit 1 (where applicable)
9	Inter-urban transit 2 (where applicable)
10	Inter-urban transit 3 (where applicable)
11	Inter-urban transit 4 (where applicable)
12	Inter-urban transit 5 (where applicable)
13	Intra-urban 2
14	Unloading
15	Stretcher-to-bed transfer
16	Connection to stationary ICU supply
17	Last contact with patient

### Statistics

Statistical analysis was performed using R 3.0.2 (R Foundation for Statistical Computing, Vienna, Austria) and SAS 9.3 (SAS Institute, Cary, NC, USA). General characteristics are presented as median, mean and standard deviation where applicable. The diagnostic value of the BIS with respect to the RASS is investigated by its sensitivity, specificity and positive predictive value. Therefore, measurements are dichotomized in conformance with literature considering a threshold of 80 for BIS values and classifying RASS values being non negative. For comparability with other publications, the relation between BIS values and the RASS was analysed by Spearman’s squared rank correlation coefficient (ρ^2^) and the Kendall rank correlation (*τ*). A moderate correlation was defined as 0.4 < *ρ*^2^ < 0.6, a strong correlation as *ρ*^2^ > 0.6. For Kendall’s tau, sufficient correlation was defined as *τ* >0.5 in accordance with previous publications. Confidence intervals were obtained by bootstrap.

## Results

**Figure 1:**
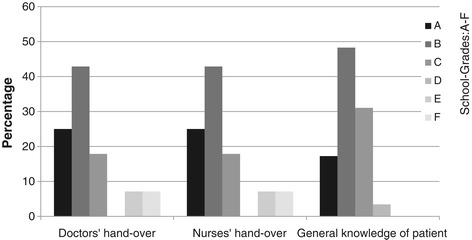
**Personal evaluation of patient handover and general knowledge about patient.** The accompanying physicians were asked to subjectively grade the quality of patient handovers on the referring wards by nurses and physicians separately. They were then asked to grade their overall knowledge about the current patient as it was gained from all available sources: handovers, study of charts, history by proxy etc. right at point when they left the ICU. Grades could range from A (very good) to F (unsatisfactory): Only in app. two thirds of cases our intensivists graded handovers or their knowledge of the patient as very good or good.

Operational data from all 30 transfers are provided in Table 3. Transport distances did vary considerably as did transfer times. The mean lead time before a mission was approximately 4 hours. However, 60% of assignments were assigned one hour or less before their start. The subjective assessment of each transport by the accompanying physician revealed that patient handovers were considered good or very good in only two thirds of cases. Complaints about episodes of restricted access to the patient were stated in 43% of cases and about restricted view in 28% of cases. In more than half of the missions the accompanying physician thought that ambient noise and vibrations (at least sometimes) negatively impacted patient care. For details see Figure 1 and Table 4.

**Table 3:** **Operational data of transfers**

	**Mean**	**Median**	**Minimum**	**Maximum**
Distance [km]	90.2	55.0	8.0	425.0
Duration [min]	114.5	90.0	45.0	300.0
Lead Time [min]	235.0	60.0	0.0	1000.0

**Table 4:** **Subjective assessment of their working environment by the accompanying intensivists**

**Question**	**Percent (number/total) responses**		
	**No-never**	**Sometimes**	**Yes-always**
Was the direct view towards the patient’s face obstructed?	72% (21/29)	28% (8/29)	0
Was the direct access to the patient impaired?	57% (16/28)	43% (12/28)	0
Was the ambient noise generally disturbing?	21% (6/29)	38% (11/29)	41% (12/29)
Had the ambient noise a negative impact on patient care?	41% (12/29)	52% (15/29)	7% (2/29)
Were ambient vibrations generally disturbing?	0	62% (18/29)	38% (11/29)
Had vibrations a negative impact on patient care?	45% (13/29)	48% (14/29)	7% (2/29)

Physicians were asked during each transfer, whether conditions of their working environment influenced their working experience or patient care.

The 8-hour average noise-exposure when travelling in the mobile ICU was 80.80 dB (A) with a peak level of 121.10 dB (A) registered during inner-city stop-and-go driving. Whole body vibrations on top of the patient’s stretcher were 0.55 m/s^2^.

The most common hypnotic agent in use was propofol (67%). Midazolam was used in 17% of patients and combinations of sedatives were rare. The combination of a sedative with an opioid however was practiced in 93% of cases. Sedation regimes are summarized in Table 5.

**Table 5:** **Sedation regimes during transfers**

**Hypnotic agent**	**Number of patients**	**Percentage**
Only propofol	21	70
Only midazolam	5	17
Propofol + midazolam	2	7
Propofol + clonidin	1	3
Only Opioid, no other hypnotics	1	3

Awakening of patients (RASS ≥0) was detected with a sensitivity of 0.97 (CI 0.89, 1.00), a specificity of 0.94 (CI 0.91, 0.96) and a positive predictive value of 0.59 (CI 0.45, 0.71) when a BIS value threshold of 80 was set (Table 6).

**Table 6:** **The number of measurements and number of corresponding patients for dichotomized BIS and RASS categories**

	**RASS ≥0**	**RASS <0**
**BIS >80**	31 measurements from 8 patients	22 measurements from 10 patients
**BIS ≤80**	1 measurement	319 measurements from all patients

Detection of patient-awakening (defined as RASS ≥0) by means of BIS monitoring (threshold of BIS >80) was highly sensitive: sensitivity 0.97 (CI 0.89, 1.00); positive predictive value 0.59 (CI 0.45, 0.71).

**Figure 2:**
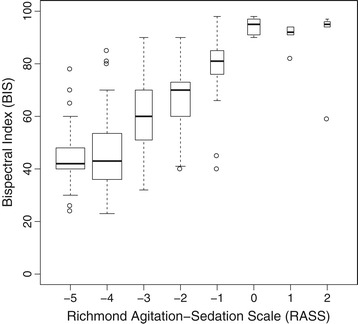
**Boxplots showing relation between bispectral index (BIS) and Richmond Agitation-Sedation Scale (RASS).** Width of boxes is proportional to the square-roots of the number of measurements. As expected the higher the RASS value, the higher the median BIS. Estimates of the median BIS for positive RASS values lack precision as only few measurements were observed (6 measurements of a RASS of 1 and 2, respectively).

The relationship of RASS and BIS is shown in Figure 2. Spearman’s rank correlation resulted to *ρ*^2^ = 0,431 (CI 0.341, 0.513) referring to a moderate correlation. The Kendall correlation coefficient was calculated as *τ* = 0.522 (CI 0.459, 0.576) representing adequate correlation.

## Discussion

Interhospital transfer of the critically ill is an independent risk factor for patient mortality and morbidity. A variety of constellations of human errors and/or equipment failure have been identified, that can lead to harmful incidents during patient transfer [12–14]. Operational data from our study demonstrates that even in a densely populated country, such as Germany, transfers may take several hours and may be assigned with little time in advance (Table 3). For these urgent referrals special preparations for special scenarios are difficult. Especially, knowledge of the patient’s history, medical condition and current procedural issues is usually narrowed down to handovers on the referring ward immediately before, or the pursuit of patient records during the transport itself. In our survey physicians rated their overall patient knowledge with grades A or B in only 65% of cases. This is only one of many reasons why the need for sufficient patient monitoring as an element of safety is recognized in current guidelines [4,15]. Recommendations for a particular monitoring of sedation levels during transfers have not been incorporated in these guidelines and to our knowledge literature does not provide a validation of objective, neurophysiological measures of brain function such as BIS in a mobile environment. The aim of our study was to investigate the value of additional BIS monitoring as a secondary method for the measurement of depth of sedation and detection of awakening during transfers in a mobile ICU.

Since its introduction into clinical practice in 2002 the Richmond agitation-sedation scale has proven reliability and validity in both sedated and non-sedated, and ventilated and non-ventilated ICU patients [8]. We therefore propose to implement the RASS for the assessment of sedation and agitation in a mobile ICU environment as well. Our study demonstrates the general feasibility of determining the RASS during transfers. Obtaining the RASS was easily introduced into routine procedures. However, there are circumstances during a transfer, when even the simple task of determining the RASS will be difficult, maybe impossible. In certain traffic conditions, the medical escort may need to be strictly seated with their seatbelts on and the patient out of reach, with no possibility of performing the RASS. In our study physicians described phases of impaired access to the patient in 43% of transports, and obstructed view of their patient in 27% of transports. Thus, movements of the patients’ hands and changes in facial expression used to generate the RASS and generally assess sedation might be undetectable. Noise levels inside the patient transport compartment were considerably high (average 85 dB (A)) with peak levels exceeding 120 dB (A). Verbal stimuli as demanded by the RASS might be disturbed and furthermore, it has to be assumed that perception of device alarms may be delayed.

Finally the use of neuromuscular blocking agents might be necessary for the safe conduct of a transfer and thus, make sedation assessment by clinical scores that observe patient reactions and movement, impossible. Consistently, the vast majority (>90%) of our transfer-service physicians would wish for an additional objective monitoring device to measure levels of sedation in those settings where subjective methods would have limited feasibility.

Of the neurophysiological methods of sedation measurement the BIS has found widespread use in anaesthesia and has proven itself a valid tool in critical care as well [9,11]. For our study we did not consider groups of patients with which either the derivation of the RASS (for example, muscle relaxation) or the BIS might be predictably faulty [16–18] (Table 1).

Correlation of measured BIS values and the RASS in a moving environment of a mobile ICU was in accordance to previous studies in a stationary ICU setting [7]. Our study demonstrates that BIS monitoring can be used as an adequate addition for sedation assessment when clinical measures are impractical.

A relevant clinical benefit is the detection of inadequate sedation and inadvertent patient awakening. In critical care, the general aim of light sedation and pre-emptive analgesia and sedation for painful and stressful procedures are integral parts of treatment [5]. Transfer in a mobile ICU may present itself as just such a painful (patient positioning and loading) and stressful (alien, loud, shaking, assumedly hostile environment) event. The concept of pre-emptive analgesia and sedation is reflected in the proclaimed aim of our physicians of a deeper sedation level for transfers (target RASS: mean -2.32; median -2). Detection of insufficiently light sedation, of awakening of a patient during a mobile ICU transfer when the patient was supposed to be sleeping through the procedure would be a meaningful application for the BIS monitor, especially in those cases mentioned above, when deriving the RASS seems impracticable.

In our study, awakening of patients (defined as RASS ≥0) was detected with a sensitivity of 0.97 (CI 0.89, 1.00) and a positive predictive value of 0.59 (CI 0.45, 0.71) for a BIS value threshold of 80. Using the BIS monitor can be a safe way to prevent unwanted awakening. The moderate positive predictive value (two false out of five alarms) seems acceptable because additional tools for the assessment of sedation (maybe even a probatory bolus of sedatives) would be available for clarification. Furthermore, low positive predictive values for device alarms are common and generally accepted as necessary to gain sufficient alarm sensitivity [19]. De Man *et al*. recently found that during induction of anaesthesia only 20% of the alarms of monitoring devices had any clinical relevance and only 11% during emergence [20].

Keeping in mind the principles of BIS and its known sources of error (Table 1), it is not so much the patient’s condition but the iatrogenic (neuromuscular blocking agents) and environmental (positioning inside truck, noise, vibrations, et cetera) factors that will define which patient will potentially benefit from the use of this device during a mobile ICU transfer.

Naturally there are limitations to our study. The BIS monitor has not been designed for use in a moving, shaking and vibrating environment. Especially, vibrations seem relevant because they may cause resonance phenomena within the electroencephalogram signal-extraction, the data source for BIS calculations. Measurements showed whole-body vibrations of up to 0.55 m/s^2^ directly underneath the patient’s thorax. It should be the manufacturer’s goal to develop algorithms to effectively filter artefacts generated in a moving environment. After implementation of such filters even stronger correlations of BIS and RASS might be achieved. Second, this was only a single-centre study and future investigations should naturally be based on a larger number of patients in a multicentre setting.

Future studies could also investigate the use of a BIS monitor in other exceptional medical environments such as air ambulances (both rotor-wing and fixed-wing) as each of these provide unique surroundings and challenges to medical care and patient assessment.

## Conclusion

Interhospital transfers of critically ill patients pose a relevant procedural risk. Assessment of levels of sedation should be performed as standard operational procedure. Sedation assessment should be considered for incorporation into clinical practice guidelines for mobile ICUs. During transfers a variety of circumstances from poor ergonomics (noise levels, patient view obstruction, poor accessibility of devices or patients) to medical conditions (relaxation) can render clinical tools for the assessment of sedation impracticable. Our study shows that objective methods of sedation measurement such as the BIS monitor may be used as a valid alternative. It revealed good correlation of BIS measures and the wel-established clinical tool of the Richmond agitation-sedation scale. The BIS monitor can be sensitive in the detection of unwanted awakening of patients during transfers. Mechanical artefacts may be responsible for the moderate positive predictive value and future studies could implement filter algorithms for the measurement of BIS in a mobile environment.

## Key messages

- We found the use of a BIS monitor to be a sensitive method for the detection of inadequate levels of sedation during interhospital transfer
- BIS monitoring may offer an alternative for those situations in a mobile ICU when neuromuscular blockage or the working environment will render clinical assessment tools such as the RASS difficult or impracticable.

## Additional file

Additional file 1:
**Study setting inside the mobile ICU: bispectral index (BIS) electrodes can be seen on the corresponding author’s forehead.** The BIS monitor is situated centrally next to the standard patient monitor. The accompanying physician was blocked from viewing the current BIS values.

## Abbreviations

BIS: bispectral index
RASS: Richmond agitation-sedation scale
WBV: whole-body vibrations
