# Supplementary figures and images for: Sedation assessment in a mobile intensive care unit: a prospective pilot-study on the relation of clinical sedation scales and the bispectral index
Source: Crit Care. 2014 Nov 24;18(6):615. doi: 10.1186/s13054-014-0615-9 (PMC4256754; doi:10.1186/s13054-014-0615-9)

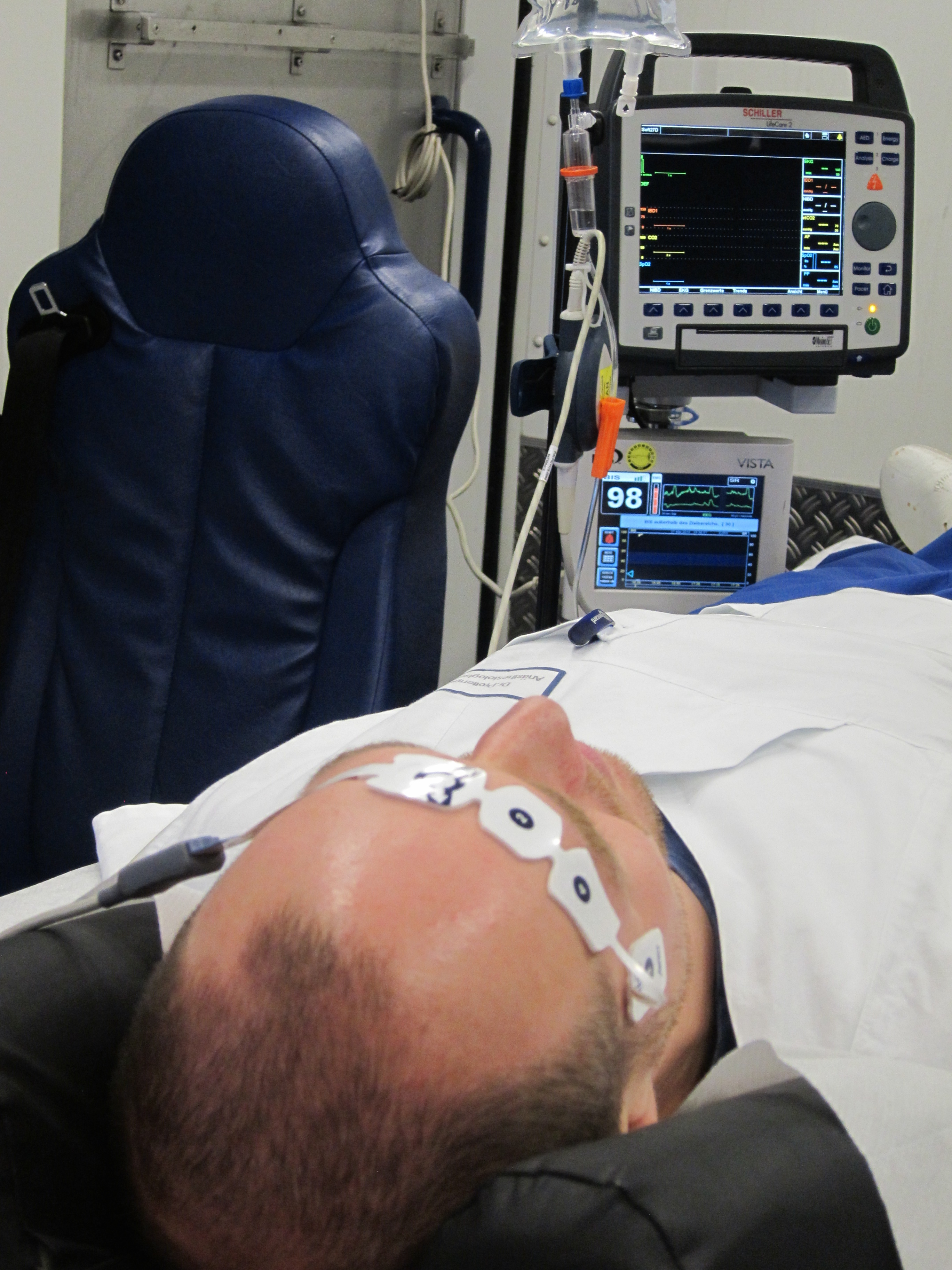

Supplement: Additional file 1: — Study setting inside the mobile ICU: bispectral index (BIS) electrodes can be seen on the corresponding author’s forehead. The BIS monitor is situated centrally next to the standard patient monitor. The accompanying physician was blocked from viewing the current BIS values. [file 13054_2014_615_MOESM1_ESM.png]
